# Risk of Diagnosed Adolescent-Onset Non-Affective Psychotic Disorder by Migration Background in British Columbia: A Retrospective Cohort Study

**DOI:** 10.1177/07067437221100351

**Published:** 2022-06-13

**Authors:** Carly Magee, Eva Oberle, Martin Guhn, Anne Gadermann, Joseph H. Puyat

**Affiliations:** 1 School of Population and Public Health (SPPH), 8166 University of British Columbia, Vancouver, British Columbia, Canada; 2 Human Early Learning Partnership (HELP), 8166 University of British Columbia, Vancouver, British Columbia, Canada; 3 Centre for Health Evaluation and Outcome Sciences (CHEOS), St. Paul’s Hospital, Vancouver, British Columbia, Canada

**Keywords:** schizophrenia spectrum and other psychotic disorders, first episode psychosis, early onset schizophrenia, immigrant health, incidence, socioeconomic status

## Abstract

**Objective:**

We recently found that the risk of diagnosed non-affective psychotic disorder between the ages of 13 and 19 was lower for immigrant adolescents compared to those without a personal or parental migration history in British Columbia (BC), Canada. In the current study, we further examined the risk for migrants compared to non-migrants by region of origin and immigrant generation (first vs. second), adjusting for several demographic factors and migration class.

**Methods:**

Administrative data were used to construct a cohort of individuals born 1990–98 and residing in South-Western BC (*N* = 193,400). Cases were identified by either one hospitalization or two outpatient physician visits with a primary diagnosis of a non-affective psychotic disorder. Poisson regression was used to estimate incidence rate ratios (IRR) of a diagnosed non-affective psychotic disorder by region of origin among first- and second-generation migrants compared to non-migrants, adjusting for sex, birth year, neighbourhood income and low family income.

**Results:**

Risk of diagnosed non-affective psychotic disorder was lower among first-generation migrants from East Asia (IRR = 0.34[95% CI: 0.25–0.46]), South-Asia (IRR = 0.47[95% CI: 0.25–0.89]) and South-East Asia (IRR = 0.55[95% CI: 0.32–0.93]) and second-generation migrants from East Asia (IRR = 0.49[95% CI: 0.35–0.69]) and South Asia (IRR = 0.52[95% CI: 0.37–0.73]), compared to non-migrants. Adjusting for migration class attenuated but did not fully explain variation in risk by region among first-generation migrants. No groups exhibited a significantly elevated risk of the diagnosed non-affective psychotic disorder compared to non-migrants.

**Conclusion:**

Findings from this study underline the complexity of the association between migration and psychotic disorders. Future research should investigate why certain groups of migrants are less likely to be diagnosed and whether there are specific sub-groups that face an elevated risk.

## Abrégé

**Objectif:**

Nous avons récemment constaté que le risque de trouble psychotique non affectif diagnostiqué entre l’âge de 13 et 19 ans était plus faible chez les adolescents immigrants comparés à ceux sans antécédents de migration personnelle ou parentale en Colombie-Britannique (CB), Canada. Dans la présente étude, nous avons examiné plus à fond le risque des migrants comparés aux non-migrants par région d’origine et génération d’immigrants (1^re^ contre 2^e^), et ajusté pour plusieurs facteurs démographiques et la classe de migration.

**Méthodes:**

Les données administratives ont servi à construire une cohorte de personnes nées entre 1990 et 1998 et résidant dans le Sud-Ouest de la CB (N = 193 400). Les cas ont été identifiés soit par une hospitalisation, soit par deux visites ambulatoires à un médecin avec un premier diagnostic de trouble psychotique non affectif. La régression de Poisson a servi à estimer les rapports des taux d’incidence (RTI) du trouble psychotique non affectif diagnostiqué par région d’origine chez les migrants de la première et de la deuxième génération comparés aux non-migrants, en ajustant pour le sexe, l’année de naissance, le revenu du quartier et le faible revenu familial.

**Résultats:**

Le risque du trouble psychotique non affectif diagnostiqué était plus faible chez les migrants de la première génération de l’Asie de l’Est (RTI = 0,34[IC à 95 % 0,25 à 0,46]), l’Asie du Sud (RTI = 0,47[IC à 95 % 0,25 à 0,89]) et l’Asie du Sud-Est (RTI = 0,55[IC à 95 % 0,32 à 0,93]) et les migrants de la deuxième génération de l’Asie de l’Est (RTI = 0,49[IC à 95 % 0,35 à 0,69]) et de l’Asie du Sud (RTI = 0,52[IC à 95 % 0,37 à 0,73]), comparativement aux non-migrants. L’ajustement pour la classe de migration atténuait mais n’expliquait pas pleinement la variation du risque par région chez les migrants de la première génération. Aucun groupe ne présentait de risque significativement élevé de trouble psychotique non affectif diagnostiqué comparativement aux non-migrants.

**Conclusion:**

Les résultats de la présente étude soulignent la complexité de l’association entre la migration et les troubles psychotiques. La recherche future devrait investiguer pourquoi certains groupes de migrants sont moins susceptibles d’être diagnostiqués et s’il y a des sous-groupes spécifiques qui courent un risque élevé.

## Introduction

Adolescent-onset psychotic disorders are associated with more severe clinical symptoms, and a longer duration of untreated psychosis compared to adult-onset cases.^1,2^ Identifying risk factors is important for planning for age-appropriate health services in diverse populations and to understand the causes of these disorders.^3,4^ Meta-analyses indicate that the incidence of psychotic disorders is 2–3 times higher for first- and second-generation migrants compared to non-migrants.^4,5^ Excess risk among migrants has been observed in both adult and adolescent age ranges^6^ and while adjusting for socioeconomic status,^5^ and appears to be greatest among migrants from Africa and the Caribbean.^4–9^ However, the majority of research has been conducted in Europe.^4,5^

Understanding associations between migration and the risk of psychotic disorders is important in Canada where almost half of the population are first- or second-generation migrants.^10^ A 2015 study of individuals aged 14–40 in Ontario^11^ found that the risk of schizophrenia/schizoaffective disorder was higher for immigrants from the Caribbean and Bermuda, and lower for immigrants from Northern and Southern Europe and East Asia, compared to non-migrants. In addition, among first-generation immigrants in Ontario, risk has been found to be higher among those who immigrated through family reunification and refugee programs compared to those who were selected based on their economic position.^12^ Similarly, an Australian study found a higher risk of psychotic disorders among immigrants from Africa but a lower risk among immigrants from South-East Asia, China and South Asia compared to non-migrants.^13^

Certain groups of immigrants may be more vulnerable to developing psychotic disorders due to exposure to stressors such as family separation, trauma, racism/discrimination and economic hardship.^14–17^ Alternatively, protective factors, such as social support and community belonging, may reduce risk among certain migrants.^18^ Finally, differences in host countries’ immigration policies and social environments may moderate associations between migration background and risk of psychotic disorders. Risk ratios have been found to be larger in the U.K. and Northern Europe and smaller in Israel and Canada.^4,19^

Research is needed to understand the level of risk of psychotic disorders among second-generation migrants in Canada and among migrants in British Columbia (BC) specifically. We recently found that the risk of any diagnosed non-affective psychotic disorder between age 13 and 19 was 38% lower among first- and second-generation immigrants combined compared to those without a family migration history in BC.^20^ Further study is needed to understand whether risk varies by migrants’ generation and region of origin. Examining heterogeneity in risk among different migrant groups may provide insight into the mechanisms underlying any observed association between migration background and risk of psychotic disorders, and identify higher risk groups in need of targeted services.

The objective of the current study was to estimate the risk of diagnosed non-affective psychotic disorder among first- and second-generation migrants by region of origin compared to non-migrants. We expected to observe patterns of risk by region of origin similar to those observed in Ontario: lower risk among migrants from East Asia and Europe and elevated incidence among migrants from the Caribbean and Bermuda.^11^ In addition, we expected that estimates of risk for second-generation migrants would fall in between estimates for first-generation migrants and non-migrants.

## Methods

### Study Cohort and Design

This study was conducted as part of a larger linked data project examining the mental health and education outcomes of migrant compared to non-migrant children and adolescents in BC.^20–22^ The study cohort included individuals born between January 1990 and March 1998, attended school in one of the ten largest districts in BC (all located in Greater Vancouver and Victoria). These districts were selected as they capture the majority (78%) of the province's immigrant population.^10^ We required that individuals were registered in the provincial health plan at age 13 (*n* = 216,173) and for a clearance period of three years (*n* = 193,495) in order to identify and exclude prevalent cases (*n* = 92).

This was a retrospective cohort study in which individuals were followed from their 13^th^ birthday until their first diagnosis of non-affective psychotic disorder, end of registration in the provincial health plan, or their 19^th^ birthday, whichever event occurred first. Registration in the health plan was determined on an annual basis (>275 days covered in a year) and was terminated by moving out of province or death.^20–22^ The observation period corresponded to the calendar years 2003–2017, depending on individuals’ birth year.

### Data Sources

This study used individual-level data from the Ministries of Education^23^ and Health,^24–26^ and the Immigration Refugees and Citizenship Canada's Permanent Resident Database.^27^ The study cohort was linked to their parents’ using records of registration in the provincial health plan. The probabilistic-deterministic linkage was performed by Population Data BC (study cohort linkage rate = 98.4%; linkage rate for parents to study cohort = 95%). The Behavioural Research Ethics Board at the University of British Columbia approved this study (UBC BREB: H10-01154).

### Case Ascertainment

Cases were defined as individuals who received a primary diagnosis in at least one hospitalization or two outpatient physician visits within two years for non-affective psychotic disorder (*ICD-10*: F20-29; *ICD-9*: 295, 297, 298). This algorithm is based on one found to have high sensitivity (94%) and moderate positive predictive value (62%).^28^ Cases who were first diagnosed at age 18 in an outpatient setting and received a subsequent diagnosis for non-affective psychotic disorder within two years were considered to have onset before age 19. To facilitate comparisons with other Canadian research, we also ran all analyses including only diagnoses for schizophrenia/schizoaffective disorder (*ICD-10*: F20 and F25; *ICD-9*: 295).^11^

### Explanatory Migration Variables

The primary exposures of interest were migrant generation and region of origin. First-generation migrants were foreign-born, whereas second-generation migrants were born in Canada to at least one migrant parent. We refer to third-generation migrants or higher as ‘non-migrants.’ In cases where the region of origin differed between parents of second-generation migrants, the mothers’ information was used. Country of origin was categorized into five broad regions and 18 sub-regions using Statistics Canada's standard classification of countries.^29^ Due to small sample sizes, we collapsed sub-regions into broad regions for all areas except for Asia. We were unable to identify second-generation migrants whose parents arrived before 1985. Migration class reflected first-generation migrants’ program of entry into Canada: economic (based on ability to contribute to the Canadian economy), family reunification (based on family sponsorship), and refugee class (based on humanitarian grounds).^30^

### Covariates

Records of registration in the provincial health plan included information on sex (female, male) and birth year (1990–92, 1993–95, 1996–98). Individuals from families that received 100% subsidy for the provincial health plan at any time during the clearance period (age 10–13) were classified as having low family income.^20^ Neighbourhood income quintile (1–5; 1 = lowest) at the start of the study (age 13) was taken from linked postal code and census information.

### Analyses

Descriptive statistics were calculated for all study variables by generation and chi-square tests were used to determine the statistical significance of any differences. Poisson regression with the logarithm of person-years of follow-up as the offset was used to estimate the crude incidence of diagnosed non-affective psychotic disorder and schizophrenia/schizoaffective disorder by generation. In addition, we calculated the incidence rate ratio (IRR) of any diagnosed non-affective psychotic disorder and schizophrenia/schizoaffective disorder specifically for (1) first-generation migrants by region of origin compared to non-migrants and (2) second-generation migrants by region of origin compared to non-migrants. For all analyses, we reported estimates adjusted for (1) sex and birth year and (2) sex, birth year, neighbourhood income and low family income. We did this to facilitate comparisons with prior Canadian research^11^ and to examine effects before and after adjusting for SES. We reported 95% confidence intervals for all estimates. Analyses were conducted in SAS software release 9.4.^31^

### Missing Data

Individuals with missing data on neighbourhood income quintile (0.85%) or low family income (2.39%) were retained in the cohort and ‘missing’ was coded as a level of each variable. Less than five individuals with missing data on sex were excluded.

### Sensitivity Analyses

We conducted sensitivity analyses with hospitalized cases only to examine whether any observed differences in risk by migration background held for more clinically severe cases. In addition, we conducted analyses with first-generation migrants only in order to assess the effect of region on the risk of diagnosed non-affective psychotic disorder while adjusting for migration class (economic, family, refugee).

## Results

### Descriptive Statistics

Table 1 shows descriptive statistics of the study sample by generation (13% first generation, 14% second generation and 73% non-migrants). The average follow-up time was 5.68, 5.82 and 5.79 years for first-, second-generation migrants and non-migrants, respectively. The proportion of first-generation migrants in our sample was similar to population estimates for the included districts (11% of individuals under age 15).^10^ The proportion of second-generation migrants in our sample was lower than in the population (25% of Canadians under age 15)^32^ likely due to our inability to detect second-generation migrants whose parents arrived before 1985.

**Table 1.** Descriptive Statistics by Generation.

	1st generation migrants (*n* = 25,442)					2nd generation migrants (*n* = 26,783)					Non-migrants (*n* = 141,175)			
	*n*	(%)	Cases NAP	Person Years		*n*	(%)	Cases NAP	Person Years		*n*	(%)	Cases NAP	Person Years
Sex														
Female	12,292	48.31	59	69,744.81		12,515	46.73	64	72,833.17		68,977	48.86	453	399,169.90
Male	13,150	51.69	68	74,797.20		14,268	53.27	108	83,039.76		72,198	51.14	657	417,853.14
Low family income														
No	12,351	48.55	71	70,621.78		16,093	60.09	97	94,396.71		103,217	73.11	613	603,819.45
Yes	11,799	46.38	<60	67,484.38		10,053	37.54	<80	58,307.40		35,253	24.97	484	198,439.14
Missing	1,292	5.08	<10	6,444.37		637	2.38	<10	3,178.63		2,705	1.92	13	14,444.70
Neighbourhood income quintile														
1 (lowest)	6,784	26.66	43	38,263.12		7,138	26.65	49	41,464.64		23,345	16.54	247	133,059.50
2	5,578	21.92	28	31,792.37		7,246	27.05	51	42,244.18		27,602	19.55	243	158,987.52
3	5,042	19.82	23	28,779.74		5,494	20.51	35	31,975.08		29,605	20.97	223	171,412.95
4	4,124	16.21	17	23,489.06		3,806	14.21	19	22,188.98		31,347	22.2	207	182,753.01
5 (highest)	3,710	14.58	<20	21,105.82		2,932	10.95	<20	17,029.06		28,003	19.84	172	163,537.52
Missing	204	0.80	<10	1,118.43		167	0.62	<10	948.56		1,273	0.9	18	7,242.10
Birth Year														
1990–92	9,432	37.07	28	53,479.44		7,666	28.62	47	44,271.15		54,173	38.37	356	312,036.48
1993–95	9,337	36.70	58	53,034.16		10,286	38.4	61	59,943.72		51,730	36.64	426	299,516.70
1996–98	6,673	26.23	41	38,020.75		8,831	32.97	64	51,661.35		35,272	24.98	328	205,388.86
Region of origin														
East Asia	12,772	50.20	43	72,289.52		6,888	25.72	35	40,294.80					
South Asia	2,074	8.15	10	11,759.58		6,673	24.92	34	39,103.78					
South-East Asia	3,044	11.96	14	17,584.27		5,155	19.25	39	30,002.10					
WC Asia and ME	1,805	7.09	16	10,234.35		825	3.08	<10	4,735.50					
Europe	2,791	10.97	17	16,020.34		3,450	11.37	22	17,600.10					
America	1,997	7.85	19	11,259.09		2,167	8.09	21	12,438.58					
Africa	730	2.87	<10	4,090.12		762	2.85	<10	4,325.87					
Oceania	229	0.90	<10	1,275.53		1,268	4.73	<10	7,388.64					
Migrant Class														
Economic	20,376	80.09	87	115,674.55										
Family	2,662	10.46	20	15,319.81										
Refugee	2,404	9.45	20	13,551.35										

*Note*. WC Asia and ME = West-Central Asia and Middle East; NAP = non-affective psychotic disorder. Cell sizes <10 are masked and the cell size of a second stratum is also masked to ensure that the small cell size cannot be determined.

Low family income was more prevalent among first- (46%) and second-generation migrants (38%) compared to non-migrants (25%) (χ^2^ = 7122.69, *p* < .0001). Similarly, a larger proportion of first- and second-generation migrants lived in low-income neighborhoods compared to non-migrants: 48% and 53% of first- and second-generation migrants and 36% of non-migrants lived in the lowest two neighbourhood income quintiles at age 13 (χ^2^ = 4808.74, *p* < .0001). Non-migrants and first-generation migrants had an approximately equal number of individuals born in 1990–92 and 1993–95 and fewer people born in 1996–98 given that our sample only included individuals born from January to March of 1998. A smaller proportion of second-generation migrants were born in 1990–92, and a larger proportion was born in 1996–98 (χ^2^ = 1147.52, *p* < .0001). Half of first-generation migrants were from East Asia. The remaining proportions were: 8% South Asia, 12% South-East Asia, 7% West-Central Asia and Middle East, 11% Europe, 8% America and 3% were from Africa. Approximately one-quarter of second-generation migrants’ parents were from East and South Asia, one-fifth were from South-East Asia, and 3% were from West-Central Asia and the Middle East. In addition, 11%, 8% and 3% were from Europe, America and Africa, respectively. The majority of first-generation migrants (80%) were economic class migrants.

### Cases

We identified *n* = 1,409 incident cases of non-affective psychotic disorder diagnosed between the ages of 13–19. The median age at index diagnosis was 17 for all groups. The majority of index diagnoses were made in outpatient settings (80% for first-generation and 75% for second-generation and non-migrants). Approximately half of the index diagnoses were for schizophrenia/schizoaffective disorder as opposed to other non-affective psychotic disorders (first-generation = 45%, second-generation = 52%, non-migrants = 46%). Using the algorithm including only diagnoses for schizophrenia/schizoaffective disorder, we identified *n* = 846 incident cases (*n* = 71 first-generation, *n* = 114 second-generation and *n* = 661 non-migrants).

The crude incidence rate of diagnosed non-affective psychotic disorder was 87.86 [95% CI: 73.80–104.50], 110.33 [95% CI: 95.00–128.1], and 135.86 [95% CI: 128.10–144.10] per 100,000 person-years for first- and second-generation migrants and non-migrants, respectively. The crude incidence rate of diagnosed schizophrenia/schizoaffective disorder was 49.08 [95% CI: 38.90–61.90], 73.06 [95% CI: 60.80–87.80], and 80.77[95% CI:74.80–87.20] per 100,000 person-years for first- and second-generation migrants and non-migrants, respectively. These estimates are higher than typical in the literature but consistent with prior Canadian studies.^3,33,34^ Higher incidence estimates in Canadian studies may be driven by differences between health systems, diagnostic practises and study design (e.g., inclusion of outpatient diagnoses in general health settings).^3^

### Risk Among First-Generation Migrants Compared to Non-Migrants by Region of Origin

Table 2 shows the estimated IRR's of diagnosed non-affective psychotic disorder and schizophrenia/schizoaffective disorder by region of origin among first- and second-generation migrants compared to non-migrants. In Model 1, the risk of diagnosed non-affective psychotic disorder was significantly lower for first-generation migrants from East Asia (IRR = 0.44[95% CI: 0.32–0.59]) and South-East Asia (IRR = 0.58[95% CI: 0.34–0.98]). After adjusting for neighbourhood income and low family income (Model 2) risk remained significantly lower among first-generation migrants from East Asia (IRR = 0.34[95% CI: 0.25–0.46]) and South-East Asia (IRR = 0.55[95% CI: 0.32–0.93]) as well as first-generation migrants from South Asia (IRR = 0.47[95% CI: 0.32–0.93)]. No other regions exhibited a statistically significantly different risk of diagnosis compared to non-migrants.

**Table 2.** Incidence Rate Ratio of Diagnosed Non-Affective Psychotic and Schizophrenia/Schizoaffective Disorder by Generation and Region, Compared to Non-Migrants (*n* = 193,400).

	Non-affective psychotic							Schizophrenia/schizoaffective						
	Model 1				Model 2			Model 1				Model 2		
	IRR	95% CI			IRR	95% CI		IRR	95% CI			IRR	95% CI	
First-generation														
Non-migrant	Reference				Reference			Reference				Reference		
East Asia	**0.44**	**0.32**	**0.59**		**0.34**	**0.25**	**0.46**	**0.44**	**0.30**	**0.66**		**0.33**	**0.23**	**0.50**
South Asia	0.61	0.33	1.13		**0.47**	**0.25**	**0.89**	**0.30**	**0.10**	**0.95**		**0.24**	**0.08**	**0.74**
South-East Asia	**0.58**	**0.34**	**0.98**		**0.55**	**0.32**	**0.93**	0.56	0.28	1.12		0.53	0.27	1.07
WC Asia and ME	1.12	0.69	1.84		0.84	0.52	1.38	1.42	0.80	2.51		1.05	0.59	1.86
Europe	0.79	0.49	1.28		0.75	0.47	1.21	0.63	0.31	1.26		0.59	0.29	1.19
America	1.22	0.78	1.92		1.11	0.70	1.74	0.98	0.51	1.89		0.88	0.46	1.70
Africa	1.24	0.59	2.61		1.13	0.54	2.36	1.50	0.62	3.60		1.34	0.56	3.24
Oceania	0.57	0.08	4.03		0.53	0.08	3.78							
Second-generation														
Non-migrant	Reference				Reference			Reference				Reference		
East Asia	**0.61**	**0.44**	**0.86**		**0.49**	**0.35**	**0.69**	0.79	0.54	1.17		**0.64**	**0.43**	**0.94**
South Asia	**0.61**	**0.43**	**0.85**		**0.52**	**0.37**	**0.73**	0.77	0.52	1.15		0.68	0.46	1.01
South-East Asia	0.93	0.67	1.27		0.78	0.57	1.08	0.92	0.60	1.39		0.79	0.52	1.19
WC Asia and ME	0.91	0.41	2.03		0.70	0.31	1.57	1.27	0.53	3.06		0.98	0.41	2.38
Europe	0.90	0.59	1.38		0.94	0.62	1.43	0.83	0.47	1.46		0.86	0.48	1.51
America	1.24	0.81	1.91		1.15	0.75	1.77	0.99	0.53	1.85		0.93	0.50	1.73
Africa	1.15	0.55	2.42		1.07	0.51	2.23	1.38	0.57	3.33		1.28	0.53	3.08
Oceania	0.78	0.39	1.57		0.79	0.39	1.57	0.99	0.44	2.20		1.01	0.45	2.25

Model 1 = adjusted for sex and birth year. Model 2 = Model 1 + adjusted for neighbourhood income and low family income. WC Asia and ME = West-Central Asia and Middle East. Estimates are bolded where 95% confidence intervals do not cross 1.0 indicating statistical significance at alpha = .05.

The observed pattern of lower risk among first-generation migrants from East, South and South-East Asia held when examining the risk of diagnosed schizophrenia/schizoaffective disorder specifically, although the effects were no longer statistically significant for migrants from South-East Asia.

### Risk Among Second-Generation Migrants Compared to Non-Migrants by Region of Origin

Risk of diagnosed non-affective psychotic disorder was significantly lower among second-generation migrants with parents from East Asia (IRR = 0.61[95% CI: 0.44–0.86]) and South Asia (IRR = 0.61[95% CI: 0.43–0.85]) in Model 1. These effects were strengthened after adjusting for neighbourhood income quintile and low family income (IRR = 0.49[95% CI: 0.35–0.69] and IRR = 0.52[95% CI: 0.37–0.73], respectively). Second-generation migrants with parents from the remaining regions did not exhibit a significantly different risk of diagnosis compared to non-migrants.

When examining the risk of diagnosed schizophrenia/schizoaffective disorder specifically, estimates of IRR were not statistically significant for any groups of second-generation migrants compared to non-migrants in Model 1. In Model 2, the risk was significantly lower for second-generation migrants with parents from East Asia compared to non-migrants (IRR = 0.64[95% CI: 0.43–0.94]).

### Sensitivity Analyses Results

We examined whether lower risk among East, South and South-East Asian migrants compared to non-migrants held when including hospitalized cases only. Due to the smaller number of hospitalized cases (*n* = 470), we combined these regions of Asia to compare IRR's when including all cases and hospitalized cases only (IRR_H_). We found that the magnitude of the effect strengthened for first-generation (IRR = 0.38[95% CI: 0.30–0.50] compared to IRR_H_ = 0.28[95% CI: 0.18–0.45]) and attenuated for second-generation (IRR = 0.58[95% CI: 0.47, 0.71] compared to IRR_H_ = 0.65[95% CI: 0.47–0.90]) Asian migrants compared to non-migrants when we restricted the case definition to hospitalized cases only.

Table 3 shows risk by region of origin and migration class among first-generation migrants only. Migrants from West-Central Asia and the Middle East, America, and Africa exhibited a significantly elevated risk of non-affective psychotic disorder compared to migrants from East Asia (IRR = 2.36–2.64). This pattern held while adjusting for migration class (Model 2b) although effects were attenuated (IRR = 2.15–2.36). Family (IRR = 1.67[95% CI: 1.02–2.73]) and refugee (IRR = 1.99[95% CI: 1.22–3.25]) class migrants exhibited an elevated risk of non-affective psychotic disorder when adjusting for sex, birth year and indicators of socioeconomic status. These effects were partially attenuated and no longer statistically significant after adjusting for the region of origin.

**Table 3.** Incidence Rate Ratio of Diagnosed Non-Affective Psychotic Disorder by Region of Origin and Migration Class Among First-Generation Immigrants Only (*n* = 25,442).

	Model 2a			Model 2b		
	IRR	95% CI		IRR	95% CI	
Region						
East Asia	Reference			Reference		
Soutd Asia	1.25	0.63	2.48	1.10	0.54	2.24
Soutd-East Asia	1.13	0.61	2.09	1.06	0.57	1.99
WC Asia and ME	**2.36**	**1.33**	**4.19**	**2.15**	**1.18**	**3.93**
Europe	1.69	0.94	3.03	1.54	0.84	2.83
America	**2.65**	**1.54**	**4.56**	**2.28**	**1.31**	**3.95**
Africa	**2.64**	**1.17**	**6.00**	**2.35**	**1.08**	**5.14**
Oceania	1.17	0.16	8.38	1.02	0.14	7.20
Migration Class						
Economic	Reference			Reference		
Family	**1.67**	**1.02**	**2.73**	1.51	0.92	2.49
Refugee	**1.99**	**1.22**	**3.25**	1.39	0.83	2.32

*Note.* Model 2a is adjusted for sex, birtd year, neighbourhood SES and family low income. Model 2b = Model2a + adjusted for region of origin and migration class. Estimates are bolded where 95% confidence intervals do not cross 1.0 indicating statistical significance at alpha = .05.

## Discussion

### Main Findings and Implications

We found that the risk of diagnosed adolescent-onset non-affective psychotic disorder was lower among first-generation migrants from East, South and South-East Asia and second-generation migrants from East and South Asia, compared to non-migrants in South-Western BC. We did not observe a statistically significant elevated risk of non-affective psychotic disorder for migrants from any region compared to non-migrants.

Our findings of lower risk of psychotic disorders among migrants from East, South and South-East Asia are consistent with a recent study conducted in Australia.^13^ Similarly, in Ontario, first-generation immigrants from East Asia had a lower risk and immigrants from South-East Asia exhibited numerically but not statistically significantly lower risk of schizophrenia/schizoaffective disorder compared to non-migrants.^11^ However, the lower risk among immigrants from South Asia compared to non-migrants was not observed in Ontario. Our findings contrast with meta-analyses of primarily European studies indicating that the risk of psychotic disorders is 50–60% higher among immigrants from Asia compared to non-migrants.^4,5^ For example, supplemental analyses from a recent Swedish study indicated that migrants from Central, North-Eastern and South-Eastern Asia all exhibited elevated risk of psychotic disorders compared to non-migrants.^8^

Lower risk of adolescent-onset psychotic disorders among Asian migrant groups compared to non-migrants in South-Western BC may be driven in part by Canada's highly selective immigration system. The majority of immigrant families are selected based on specific education and employment experience, and official language ability (i.e., English or French).^35^ Research indicates that proficiency in the host country language is associated with a lower risk of psychotic disorders among immigrants.^36^ In addition, numerous studies have found that first- and second-generation immigrant youth have lower rates of mental and physical disorders and greater academic achievement compared to non-migrants.^37–39^ Our finding that first-generation migrants selected based on their economic position had a lower risk of adolescent-onset psychotic disorder compared to those selected through family reunification and refugee programs is consistent with research in Ontario and demonstrates the impact of selection biases on the risk of psychotic disorder among migrant groups in Canada.^12^ However, migration class did not fully explain the observed differences in risk by region of origin in our study.

The large and established Asian population in South-Western BC is also likely protective for Asian immigrant youth in particular.^10,11^ Similar to Australia, top source countries for immigration to Canada include China, India and the Philippines.^10,40^ People of East, South and South-East Asian heritage comprise 23%, 11% and 8% of the total population in South-Western BC.^10^ In comparison, Asian migrant and ethnic groups comprise a much smaller proportion of the total population in European countries (5–8% in total).^41,42^ Neighbourhood ethnic density has been associated with a lower risk of psychotic disorder among migrants, potentially through reducing exposure to discrimination and facilitating connection with others who share common languages, cultural norms and values.^43,44^ In addition, psychosocial assets such as ethnic/cultural identity, community and family ties may serve as protective factors for Asian immigrant youth in South-Western BC.^45–47^ Further research is needed to understand how these factors may relate to the risk of psychotic disorders among migrant youth in BC.

Lower diagnosed incidence of adolescent-onset psychotic disorders among Asian migrants compared to non-migrants may also have resulted from lower detection of psychotic disorders in the health system in these groups. Numerous studies have found that Asian migrants under-utilize mental health services compared to non-migrants, although these differences appear to be less pronounced for psychotic disorders compared to other mental disorders.^48–51^ If Asian migrant adolescents were less likely to access health services despite an equal or higher risk of psychotic disorders, we may expect that the risk of reaching a crisis point requiring hospitalization would be higher for this group. However, we found that lower risk for first- and second-generation Asian migrants compared to non-migrants held even while examining hospitalized cases only, although the magnitude of the effect was attenuated for second-generation Asian migrants. Similarly, effects were attenuated for second-generation Asian migrants when including only schizophrenia/schizoaffective diagnoses. These findings suggest that less clinically severe cases of adolescent-onset psychotic disorder may be under-detected in the health system for second-generation Asian migrants in particular. Future research should investigate potential barriers to accessing early psychosis services among different migrant groups in BC.

### Strengths and Limitations

Strengths of this study included the population-based sample and the use of linked administrative data from multiple sources. This allowed us to examine the distribution of the risk of adolescent-onset psychotic disorders in various migrant subgroups compared to non-migrants, adjusting for indicators of socioeconomic status at both the neighbourhood and family level. In addition, immigration records from both the cohort and their parents allowed us to assess the risk of diagnosis among first- and second-generation migrants.

This study was limited by the fact that case ascertainment depended on diagnosis in the health system.^52^ We may have missed cases who accessed other forms of care (e.g., community supports, private counselling) and there may have been systematic differences in physician tendencies to diagnose psychotic disorder by ethnicity or migration background. However, existing research suggests that Asian migrants are more commonly over-diagnosed with psychotic disorders (rather than under-diagnosed) which would have biased the observed effects towards the null.^53^ Further research is needed to understand whether the sensitivity and specificity of administrative data algorithms to detect psychotic disorders varies by ethnicity or immigration background in Canada.

There was likely residual confounding by household-level income in this study given that our measure (1) only distinguished between the lowest income families and everyone else and (2) required that families opt into subsidies for BC's health plan (3) was measured only at the start of the study period. Effects by migration background were also likely under-estimated due to our inability to identify second-generation migrants whose parents migrated before 1985, the majority of whom would have been from Europe and Asia.^54^ In addition, the small number of refugee and family class migrants and migrants from specific areas (e.g., Africa, America, Europe, Oceania) in our sample limited our power to estimate risk in these groups. Moreover, the included regions were extremely broad and may contain sub-groups with elevated risk. ^11^ Further research with larger sample sizes should investigate whether specific immigrant and minority groups have a higher risk of adolescent-onset psychotic disorders in BC.

## Conclusion

Findings from the study indicate that first-generation migrants from East, South and South-East Asia and second-generation migrants from East and South Asia are less likely to be diagnosed with non-affective psychotic disorder during adolescence compared to non-migrants in South-Western BC. However, these effects were not observed among migrants from other world regions. These findings underline the fact that the association between migration and psychosis is complex. It does not appear that the experience of migration alone increases risk of psychotic disorders. Selective pressures of immigration systems, the social context of host countries, and the social position of migrants within host countries all likely influence associations between migration and risk of psychotic disorders.
